# Evaluating stereoacuity with 3D shutter glasses technology

**DOI:** 10.1186/s12886-016-0223-3

**Published:** 2016-04-26

**Authors:** Huang Wu, Han Jin, Ying Sun, Yang Wang, Min Ge, Yang Chen, Yunfeng Chi

**Affiliations:** Department of Optometry, Second Hospital of Jilin University, No. 218, Ziqiang Street, Nanguan District, Changchun, 130041 China; University of Virginia, Charlottesville, VA USA; Department of Ultrasonography, First Hospital of Jilin University, Changchun, China

**Keywords:** Stereopsis, Shutter glasses, Horizontal disparity

## Abstract

**Background:**

To determine the stereoacuity threshold with a 3D laptop equipped with 3D shutter glasses, and to evaluate the effect of different shape and size of test symbols and different type of disparities to stereoacuity.

**Methods:**

Thirty subjects with a visual acuity in each eye of at least 0 logMAR and a stereoacuity of at least 32 arcsec (as assessed in Fly Stereo Acuity Test) were recruited. Three target symbols—tumbling "E", tumbling "C", and "□"—were displayed, each with six different sizes representing a visual acuity ranging from 0.5 to 0 logMAR when tested at 4.1 m, and with both crossed and uncrossed disparities. Two test systems were designed - fixed distance of 4.1 m and one for variable distance. The former has disparities ranging from 10 to 1000 arcsec. Each subject completed 36 trials to investigate the effect of different symbol sizes and shapes, and disparity types on stereoacuity. In the variable distance system, each subject was tested 12 times for the same purposes, both proximally and distally (the point where the 3D effect just appears and where it just disappears respectively), and the mean value was calculated from the mean proximal and distal distances.

**Results:**

No significant difference was found among the groups in the fixed distance test system (Kruskal-Wallis test; *Chi-square* = 29.844, *P* = 0.715). Similarly, no significant difference was found in the variable distance system (Kruskal-Wallis test; proximal: *Chi-square* = 5.687, *P* = 0.338; distal: *Chi-square* = 5.898, *P* = 0.316; mean: *Chi-square* = 6.152, *P* = 0.292).

**Conclusions:**

Evaluating stereoacuity using this measurement system was convenient and effective. Changes in target shape and size and disparity types had no significant effect on stereoacuity. It would be helpful to choose optimal targets according to different purposes using computer-assisted 3D measurements.

**Electronic supplementary material:**

The online version of this article (doi:10.1186/s12886-016-0223-3) contains supplementary material, which is available to authorized users.

## Background

Stereopsis is a higher function of two-eye coordination, which enables a precise judgment of distance. Generally, it is possible to determine distance with monocular vision from looming, motion parallax, and pictorial depth cues such as occlusion, perspective, texture gradients, relative size, and shadows. However, the most precise distance determination is achieved through stereopsis, which is a specific type of binocular depth perception resulting from the horizontal separation of the two eyes and the subsequent ability to recognize retinal disparity [1]. Stereopsis is important for carrying out specific tasks, including better motor control and quicker and more accurate cognitive information [2]. Stereopsis is quantified as the *minimum geometric disparity* that elicits the perception of depth termed stereoacuity, measured in seconds of arc (arcsec) [1]. Stereoacuity is sometimes measured from a distance of 3–6 m, e.g., the Frisby–Davis distance stereotest [3–9]. The majority of measurements are performed at a closer range, usually 0.4 m, with e.g., the Titmus Fly Test using polarization technology [9–14], and the TNO Stereoacuity Test using red and green glasses [9, 10, 15–19]. These traditional methods are widely used in clinical practice and experimental research. Since the pattern and number of test pictures are relatively fixed, it can be overwhelming and painstakingly slow to study a large number of influencing factors for stereopsis. With the development and advances in three-dimensional (3D) computer technology over recent years, researchers have designed new stereopsis tests with modern techniques [20–25]. At this point, no definite conclusions have been drawn on whether the size or the shape of the target would affect the test result and how to choose an ideal symbol to explore stereopsis on the display screen. Thus, we established a stereoacuity measurement system using 3D shutter glasses technology and designed different test targets in order to assess the effects of different factors on stereoacuity. We also applied two methods to evaluate those factors: the fixed distance test and the variable distance test. The aim of our study was to investigate the effect of different target shapes and sizes on stereoacuity, and it would be the condition to choose optimal targets according to different purposes with computer-aided 3D measurement.

## Methods

### Subjects

The study was conducted at the Second Hospital of Jilin University in China. A total of 30 subjects, aged 20–28 (22.9 ± 2.4) years, were included, comprising 11 males and 19 females. The refractive status of the right eyes were as follows: spherical error +0.50 DS to −4.00 DS (−1.42 ± 1.16 DS), cylinder error 0 to −1.00 DC (−0.64 ± 0.18 DC), spherical equivalent +0.50 DS to −4.00 DS (−1.48 ± 1.14 DS). The refractive status of the left eyes were as follows: spherical error +0.50 DS to −4.00 DS (−1.50 ± 1.20 DS), cylinder error 0 to −1.75 DC (−0.92 ± 0.47 DC), spherical equivalent +0.50 DS to −4.00 DS (−1.59 ± 1.14 DS). None of the study participants had severe ametropia, amblyopia [26], strabismus [27], or anisometropia [28]. The minimum corrected visual acuity of each eye was 0 logMAR. The stereoacuity, as measured by the Fly Stereo Acuity Test (Vision Assessment Corporation, Elk Grove Village, IL, USA), was at least 32 arcsec.

### Computer system

**Fig. 1:**
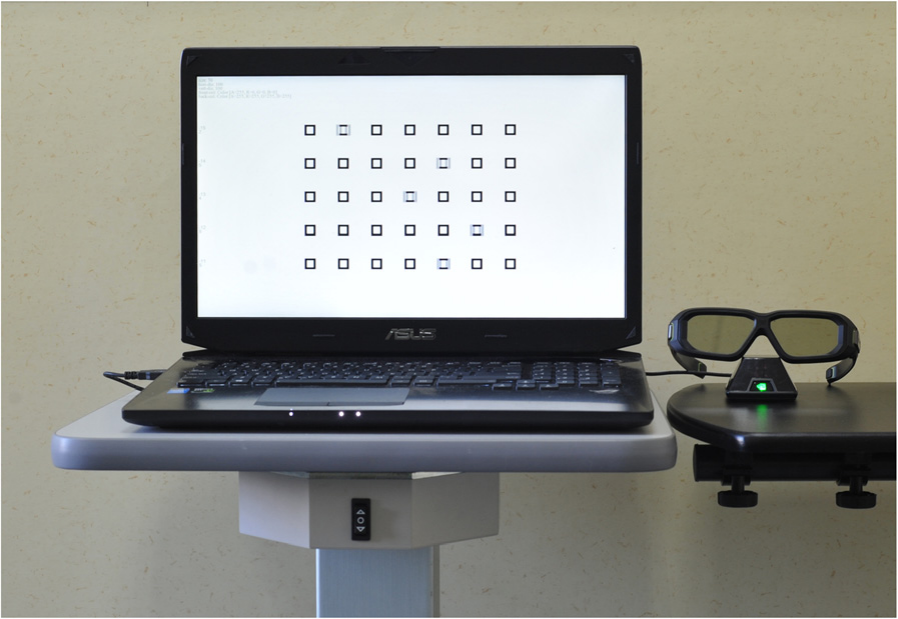
Photograph showing the 3D laptop (ASUS G750Y47JX) equipped with NVidia 3D Vision 2 Wireless Glasses Kit (Expressway Santa Clara, CA, USA)

We designed a notebook computer system, using a laptop (ASUS G750Y47JX, 17.3" 16:9 full HD 3D (1920 × 1080 120 Hz)) running Windows 8.1 and NVidia 3D Vision 2 Wireless Glasses Kit (Expressway Santa Clara, CA, USA). The NVidia 3D Vision Photo Viewer Software was used to view 3D pictures (Fig. 1).

### Test targets

**Fig. 2:**
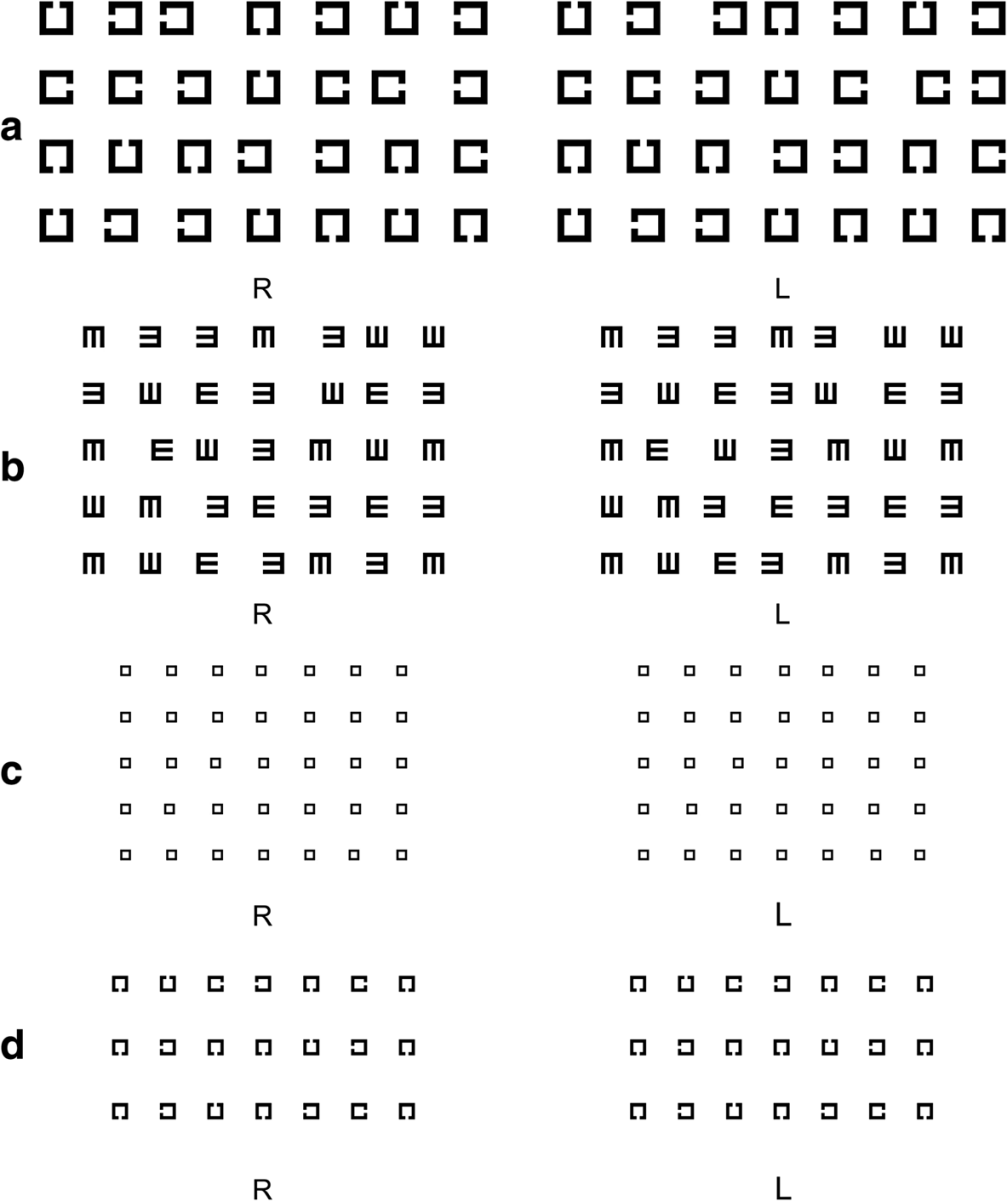
Illustrations of the stereoacuity test system. **a** Example of the fixed distance test system. The shape of the symbol is c; the size of the symbol is 95 × 95 pixels; the type of disparity is crossed, and the grade of the test is first (four lines represented the disparity of 100 pixels, 75 pixels, 50 pixels and 25 pixels from top to bottom). The stereo targets are line 1–3, line 2–6, line 3–4, and line4-2. **b** Example of the fixed distance test system. The shape of the symbol is E; the size of the symbol is 60 × 60 pixels; the type of disparity is uncrossed, and the grade of the test is second, page 2 (five lines represented the disparity of 75 pixels, 70 pixels, 65 pixels, 60 pixels and 55 pixels from top to bottom). The stereo targets are line 1–5, line 2–5, line 3–2, line4-3, and line 5–4. **c** Example of the fixed distance test system. The shape of the symbol is □; the size of the symbol is 30 × 30 pixels; the type of disparity is crossed, and the grade of the test is third, page 3 (five lines represented the disparity of 15 pixels, 14 pixels, 13 pixels, 12 pixels and 11 pixels from top to bottom). The stereo targets are line 1–4, line 2–4, line 3–3, line4-2, and line 5–6. **d** Example of the variable distance test system. The shape of the symbol is C; the size of the symbol is 45 × 45 pixels; the type of disparity is crossed. The reference target in the top line is no.4 with 3 pixels disparity, while in the bottom line is no.3 with 2 pixels disparity. The test stereo target in the middle line is no.5 with 1 pixels disparity

A program written in C# produced all test targets. Three types of symbols were drawn: tumbling "E", tumbling "C", and "□" (Fig. 2). Each type of symbol had six different sizes, containing strokes that were 19, 15, 12, 9, 7, and 6 pixels (px) wide. Hence, the complete symbols had sizes of 95 × 95, 75 × 75, 60 × 60, 45 × 45, 35 × 35, and 30 × 30 px. All symbols were set at 100 px apart, both horizontally and vertically. The symbols with 3D effect were slightly different; the perceived images of the symbols were at the midpoint of the L and R images, which was 100 px away from other symbols. The test distance was set at 4.1 m, and the symbol sizes represented 0.5, 0.4, 0.3, 0.2, 0.1, and 0 logMAR respectively, under these conditions.

Seven symbols were drawn in a line. One symbol, which was the stereo target, was chosen randomly from the five middle symbols and rendered with 3D depth. The stereo target consisted of two component images, each of which was visible with one eye only as the 3D shutter glasses blocked the other eye. The distance between the two objects represented the horizontal disparity. Crossed disparity occurred when both eyes saw an image on the opposite side. If the right image of the symbol was seen by the left eye, and the left image was seen by the right eye, the target would be perceived as a single image through the 3D shutter glasses. It would appear in a different plane that was closer to the observer, compared to the other symbols on the same line. Conversely, if the right side of the symbol was seen by the right eye, and the left side was seen by the left eye, the target would be seen denting into the screen, compared to the other symbols, and this is called uncrossed disparity. The depth of extrusion of the stereo target was determined by the degree of disparity. Thus the larger the disparity, the more visible the stereo perception would be. When the disparity decreased to a certain threshold, the observer would not be able to distinguish between the stereo target and the other symbols. The aim of our test was to detect the minimum disparity at which the study participant could still see the stereo target.

### The fixed distance test system

In our study, the test distance was set at 4.1 m, and hence 1 px corresponded to a disparity of 10 arcsec. The fixed distance test system contained three grades of difficulty. The first grade consisted of a single page that contained four lines, with disparities of 100, 75, 50, and 25 px (range step 25 px), respectively. The second grade consisted of four pages, each containing five lines that represented the following disparities: 80 to 100, 55 to 75, 30 to 50, and 5 to 25 px (range step 5 px), respectively. In the third grade, each page contained five lines with disparities ranging from 1 to 100 px (range step 1px), and the distance between the lines and the objects were all 100 px (Fig. 2). The purpose of the pages was to find the range that contained the threshold disparity at which the subject just failed to identify the stereo target; the grades presented increasing levels of difficulty (higher precision required), such that the range was narrowed down to 1 px.

The subject sat at a distance of 4.1 m away from the monitor. The first grade, which had the largest range, was used as a teaching picture tool. The participant was trained to identify different targets from the seven symbols in a line through 3D shutter glasses. For crossed disparity, the symbol appeared to come out of the screen and closer to the subject, compared to the other symbols on the same line. For uncrossed disparity, the symbol appeared to go into the screen and further away from the subject, compared to the other symbols on the same line.

First, the subject was asked to distinguish the stereo target, line by line, on the first grade page. If the subject found the stereo target on the third line but could not point out the difference on the last line, it meant that their stereoacuity was between 50 and 25 px (excluding 25 px). Then, the subject was asked to repeat the identification using a page from the second grade; in this case, the page of 30 to 50 px. If the subject found the target symbol on the fourth line, but not on the fifth line, it meant their stereoacuity was between 35 and 30 px (excluding 30 px). Then the third grade test page of 35 to 31 px was displayed. If the subject pointed out the stereo target on the second line, but not on the third line, the perceptible disparity for that subject was 34 px, indicating that their stereoacuity was 340 arcsec. Using this progressive approach, the stereoacuity of each subject was determined.

The subjects’ stereoacuity were all reasonably good, as assessed by the Fly Stereo Acuity Test, so they should be able to distinguish a difference on the first grade test page easily. After familiarizing with the feel of seeing the stereo target at relatively large disparities, subjects were shown the last test page, which contained disparities from 5 to 1 px, representing stereoacuity from 50 to 10 arcsec.

We designed 36 sets of test patterns, which were composed of three symbol types containing crossed and uncrossed disparity, with each having six sizes, corresponding to 0.5, 0.4, 0.3, 0.2, 0.1, and 0 logMAR visual acuities. We examined the subjects using those 36 pattern sets randomly, with a 3-min break after every 12 tests.

### The variable distance test system

The subject was asked to sit at different distances, looking at a page of three lines containing the test symbol. The size of the symbol was 45 px. The disparity of the stereo target was 3 px in the top line, 2 px in the bottom line, and 1 px in the middle line. The distance between the lines and the symbols were all 100 px (Fig. 2).

First, the subject was trained to identify the stereo target at a relatively near distance to the display. Then the subject was moved further away from the screen, until they could not detect the stereo target on the middle line. Then the participant was asked to slowly move towards the monitor, until they could just point out the target symbol, and the distance from the eyes to the display was measured and recorded with a laser rangefinder (GLM 30 Professional, BOSCH, Milton Keynes, UK). Then the subject was asked to slowly move away from the monitor, until they could no longer point out the target symbol, and the distance from the eyes to the display was measured. The stereoacuity was calculated using the formula containing the distance and disparity: dγ = aΔd/d^2^. The first result represented the proximal stereoacuity, and the second result the distal stereoacuity. The mean value, calculated from the mean distance between proximal and distal results, is the stereopsis of the subject.

Six test pattern sets, composed of three types of crossed and uncrossed symbols, were drawn and used to test the subjects. The test was repeated three times, with a 3-min break between each trial.

### Statistics

All data were processed using PASW Statistic 18.0 (IBM SPSS Inc.). We used Shapiro-Wilk to explore the distribution form of the data first. If the data satisfied normal distribution patterns, parametric tests were used for statistical data analysis, and we applied one-way ANOVA tests to analyze the differences among those groups. If the data were not normally distributed, non- parametric tests were carried out. We used the Kruskal-Wallis test (a test for several independent samples in non-parametric tests) to analyze the differences among those groups.

## Results

### The fixed distance test system

Each subject recruited in our study completed 36 tests for the effect of different symbol sizes and shapes, and different types of disparity on stereoacuity. The Shapiro-Wilk test showed that no group of data satisfied normal distribution (*P* < 0.001), so we used median (M) and quartile range (QR) to describe the concentrations and discrete trends shown in Table 1. Using the Kruskal-Wallis test, no significant difference was found among the 36 groups (*Chi-square* = 29.844, *P* = 0.715). These results showed that stereoacuity was consistent among the study population, regardless of the size of the symbols (ranging from 0.5 to 0 logMAR), the symbol shape ("C", "E", or "□"), and the types of disparity (either crossed or uncrossed).

**Table 1:** Stereoacuity M (QR) in arcsec of 36 groups tested with the fixed distance system

Target	C-crossed- 0.5logMAR	C-crossed- 0.4logMAR	C-crossed- 0.3logMAR	C-crossed- 0.2logMAR	C-crossed- 0.1logMAR	C-crossed- 0logMAR
Stereoacuity	20 (13)	20 (10)	15 (10)	15 (10)	10 (13)	20 (20)
Target	C-uncrossed- 0.5logMAR	C-uncrossed- 0.4logMAR	C-uncrossed- 0.3logMAR	C-uncrossed- 0.2logMAR	C-uncrossed- 0.1logMAR	C-uncrossed- 0logMAR
Stereoacuity	20 (10)	10 (10)	10 (10)	10 (10)	10 (10)	20 (10)
Target	E-crossed- 0.5logMAR	E-crossed- 0.4logMAR	E-crossed- 0.3logMAR	E-crossed- 0.2logMAR	E-crossed- 0.1logMAR	E-crossed- 0logMAR
Stereoacuity	20 (10)	2p0 (10)	20 (10)	10 (10)	15 (10)	10 (10)
Target	E-uncrossed- 0.5logMAR	E-uncrossed- 0.4logMAR	E-uncrossed- 0.3logMAR	E-uncrossed- 0.2logMAR	E-uncrossed- 0.1logMAR	E-uncrossed- 0logMAR
Stereoacuity	10 (13)	20 (10)	20 (10)	15 (10)	20 (10)	10 (13)
Target	□-crossed- 0.5logMAR	□-crossed- 0.4logMAR	□-crossed- 0.3logMAR	□-crossed- 0.2logMAR	□-crossed- 0.1logMAR	□-crossed- 0logMAR
Stereoacuity	15 (10)	10 (10)	10 (10)	10 (10)	10 (10)	10 (10)
Target	□-uncrossed- 0.5logMAR	□-uncrossed- 0.4logMAR	□-uncrossed- 0.3logMAR	□-uncrossed- 0.2logMAR	□-uncrossed- 0.1logMAR	□-uncrossed- 0logMAR
Stereoacuity	10 (10)	20 (10)	10 (10)	15 (10)	10 (10)	10 (10)

### The variable distance test system

This test system had some challenges for both the examiner and the subjects. Based on an easy first and difficult afterwards principle, we always tested using the fixed distance system first followed by the variable distance test system. The examiner had to measure the distance from the eye plane of the subject to the screen plane of the laptop with a laser rangefinder. In our study, all subjects completed the test smoothly, as they had already experienced the fixed distance system and thus understood the procedure correctly. Since no significant differences among the different symbol sizes were obtained from the fixed distance test system, we chose 45 px which represented 0.2 logMAR at 4.1 m as the standard size of the test symbol. When the subject moved towards the screen within 4.1 m, the relative size of the targets became slightly bigger than 0.2 logMAR. Conversely, when moving away from the screen beyond 4.1 m, the relative size of the targets became slightly smaller than 0.2 logMAR. However, the test results were not affected. The distance measurement error was not a significant influencing factor, i.e., if the right position was at 4.1 m, but the actual measured distance was at 3.1 m, the result error was only 3 arcsec, and when the actual measured distance was at 5.1 m, the result error was only 2 arcsec.

In this part of our study, we tested each subject 12 times for the effect of the different symbols or the different types of disparity on stereoacuity both proximally and distally (the distance where the target just appears and where the 3D effect just disappears). We calculated the mean value as the representative stereoacuity threshold of an individual in our test system. Note that the mean value was not the mean of the proximal and distal stereoacuity values, but was calculated from the mean proximal and distal distances.

All 18 groups of data showed skewed distribution under the Shapiro-Wilk test (*P* < 0.01). The Kruskal-Wallis test identified no significant difference among the six groups (proximal: *Chi-square* = 5.687, *P* = 0.338; distal: *Chi-square* = 5.898, *P* = 0.316; mean: *Chinsquare* = 6.152, *P* = 0.292). The data is presented in Table 2. Our findings indicated that the shape of the targets ("C", "E", or "□") and the types of disparity (crossed or uncrossed) were not significant factors affecting stereoacuity.

**Table 2:** Stereoacuity M (QR) in arcsec of 18 groups tested with the variable distance system

Disparity	C- crossed	C-uncrossed	E-crossed	E-uncrossed	□-crossed	□-uncrossed
proximal	10.25 (3.40)	10.15 (4.13)	11.70 (4.82)	9.80 (4.03)	9.90 (4.20)	9.90 (3.97)
distal	9.30 (3.00)	8.65 (3.38)	10.00 (3.30)	8.50 (3.93)	8.65 (3.73)	8.50 (3.42)
mean	9.65 (2.98)	9.15 (3.75)	10.80 (4.10)	9.05 (3.80)	9.15 (3.95)	8.95 (3.60)

## Discussion

Stereopsis is the ability to precisely judge distance, and its classification is rather complicated, depending on different definitions, such as fine and coarse, or local and global. With the development of information technology, 3D displays can be driven by computers. Thus a 3D polarization technique was used to evaluate stereopsis, as described by Kim & Yang et al. [25] who developed a contour-based stereotest using a 46-in. 3D monitor, which can measure 5000 to 20 arcsec of distance stereopsis.

What we use now is a kind of mature 3D display called active shutter glasses, which is widely used to view 3D movies or to play 3D video games [29–32]. The core components of the active shutter 3D system are liquid crystal shutter glasses and a monitor with a high refresh rate. The principle of this technique is that the glasses contain a liquid crystal layer which turns immediately from opaque to transparent when a voltage is applied, and the alternately block each of the two eyes, such that they receive different images. A high refresh rate monitor of 120 Hz, equipped with a time controller, can display two different images alternately, synchronizing with the shutter glasses; therefore, each eye receives a different image. Because the transition from unblocking to blocking is so fast, the observer still receives 60 frames per second in each eye and therefore could perceive the displayed image as if looking at a regular screen; hence the binocular vision is divided. When the computer alternates between the two components of a 3D image, subjects will perceive a 3D image.

The test 3D images (in JPS format) were produced using a program written in C#, and share a similar file format with JPEG images but consist of two image components divided by a vertical line through the center. JPS 3D images can be displayed easily with NVidia 3D Vision Photo Viewer by a skilled clinician. In our experience, this 3D technique was applied successfully, in theory and practice, to evaluate stereopsis in subjects with normal vision. We tested stereoacuity using our system compared with the Fly Stereo Acuity Test, and the two tests showed reconciled results [33]. Our findings show that both the fixed and variable distance 3D measurement systems are suitable for people with normal vision.

The advantages of this 3D system are precision and flexibility. Thus, the binocular disparities can be changed with a minimum step size of 10 arcsec in the fixed distance measurement system; in the variable distance system, as the disparity changes continuously with distance, the precision is only restricted by the accuracy of the distance measurement. In the 3D system, any forms of symbols, images, or texts can be applied or manipulated conveniently for different purposes or different groups of subjects. However, the disadvantages of the system are that subjects differ in their reactions, and therefore some people need to be familiarized with the system first, and the longitudinal space should be sufficiently long, especially for the variable distance test method.

The reason we explored different symbols in this study, including "C", "E", and "□", was to find an ideal symbol to use in future stereoacuity research. The tumbling "E" is used most commonly as a visual acuity chart test object in China. It has become the standard symbol for the measurement of visual acuity in Chinese clinics, so it may be helpful to study stereoacuity alongside visual acuity. Some subjects reported that, when the opening direction of a tumbling E target pointed to the left or right, they felt slightly confused, maybe when it appeared at first glance, compared to the opening direction pointing up or down; however, the subjects recovered promptly. Therefore, we designed another square-shaped target "□", based on the circle symbols used in the Titmus and Fly Stereo Acuity Tests. We used a square, rather than a circular, shaped target, due to the limitation of the display method on the computer where, for a high-resolution picture, the transition of the border of a circle cannot be as perfect as that of a square because of the pixel arrangement on the screen. However, the results showed no significant difference between the tumbling "E" and the "□". In order to preserve the function of assessment of the visual acuity and reduce the horizontal disparity area difference, we designed another symbol, the tumbling "C". It is like the Landolt C, but with square, not circular, strokes of the target, and four instead of eight pointing directions like the tumbling "E". No significant difference was found among the three symbols in our study. In addition, we drew symbols from 30 × 30 px to 90 × 90 px, which were all in the range of clear recognition. In this part of the experiment, we confirmed that a target size larger than the recognition resolution was not an effective influencing factor in the evaluation of stereopsis.

Whether or not a significant difference exists between the thresholds for crossed and uncrossed disparity to see has been controversial in the literature. Woo and Sillanpaa found a lower threshold for cross disparities than for uncrossed disparities from stereoscopic tests of 30 subjects [34], and similar results were reported by Grabowska [35]. However, the opposite observation was made by Larson who investigated the difference in stereoacuity between crossed and uncrossed disparities in 15 subjects using Frisby and TNO tests [36]. In addition, Jaschinski and Schroth examined 11 subjects with normal vision and a stereo resolution of less than 100 arcsec, and only a minority of them showed a difference between crossed and uncrossed disparities [37]. In our study, no significant difference was found between crossed and uncrossed disparities.

The variable distance test system has a better theoretical design for its relatively simple test target system and smooth change. The main problem with this system is that unlike the fixed distance system, when subjects report the disappearing of 3D effect, they already know which symbol is the target. To test the proximal stereoacuity, the subjects have to figure out the different target while moving towards the screen slowly without any psychological hint. To test the distal stereoacuity, the subjects are asked to report the specific moment when the different target symbol disappears while moving away from the screen slowly, also without any psychological hints. It is difficult to avoid any psychological influences for subjects who are not well trained, and the subject can cheat easily if they want to gain a good score. The variable distance test system may not be an effective tool to measure stereoacuity in the clinic, but it may be a powerful research tool to study the influencing factors of stereoacuity where subjects are highly trained medical staff who understand the aim of the test system. In our study, subjects reported well using the variable distance system; the distance between the proximal and distal end was 0.7 ± 0.3 m, and the difference between the proximal and distal stereoacuity was 2.3 ± 2.5 arcsec. We calculated the mean stereoacuity by taking the mean value calculated from the proximal and distal distances, and then using the corresponding mean stereoacuity as the stereopsis of the subject. The method was originally developed in the Howard-Dolman Peg Test, which used the just noticeable difference (JND) threshold [38]. It is practically meaningful, because the fluctuation range of the proximal and distal stereoacuity is much smaller than the smallest step size of common clinical stereopsis evaluation methods.

For future investigations, we would study the effects of other factors on stereoacuity in subjects with normal vision, including brightness, contrast, color, crowdedness, etc. Although these studies can be difficult to carry out using traditional measurement tools, they can be conducted easily using our 3D measurement system. In addition, we would study the performance of subjects with abnormal stereoacuity such as those who have amblyopia under different circumstances.

## Conclusions

Evaluating stereoacuity with 3D shutter glasses technology was convenient and effective in both the fixed distance and variable distance test systems. Changes in target shape and size and disparity types had no significant effect on stereoacuity and it would be helpful to choose symbols according to different purpose with computer-aided 3D measurement in future.

## Ethics approval and consent to participate

All participants gave their informed written consent before taking part. The research protocol observed the tenets of the Declaration of Helsinki and was approved by the ethics committee of the Second Hospital of Jilin University (No.2015-45).

## Consent for publication

Not applicable for this study.

## Availability of data and materials

All the raw data of this article is shown in Additional file 1: Raw data (stereoacuity). xls. The data of personal identity information will not be made available in order to protect the participants’ privacy.

## Additional file

Additional file 1: Raw data (stereoacuity). (XLS 75 kb)

## Abbreviations

3D: Three-dimension
ANOVA: Analysis of Variance
arcsec: Seconds of arc
DC: cylindrical dioptor
DS: spherical dioptor
HD: high definition
JPEG: joint photographic experts group
MAR: minimum angle of resolution
Px: Pixel

## References

[1] Daum KM, McCormack GI. Fusion and Binocularity. In Benjamin, WJ (ed). Borish's Clinical Refraction. 2nd ed. St. Louis: Butterworth–Heinemann Elsevier; 2006:145-191.

[2] Gray R, Regan D (1998). Accuracy of estimating time to collision using binocular and monocular information A. Vision Res.

[3] Eom Y, Song JS, Ahn SE, Kang SY, Suh YW, Oh J, Kim SH, Kim HM (2013). Effects of interpupillary distance on stereoacuity: the Frisby Davis distance stereotest versus a 3-dimensional distance stereotest. Jpn J Ophthalmol.

[4] Singman EL, Matta NS, Silbert DI, Tian J (2013). Comparison of the INNOVA Visual Acuity System Stereotest with the Frisby-Davis 2 Stereotest for the Evaluation of Distance Stereoacuity. Binocul Vis Strabolog Q Simms Romano.

[5] Saxena R, Kakkar A, Menon V, Sharma P, Phuljhele S (2011). Evaluation of factors influencing distance stereoacuity on Frisby-Davis Distance Test (FD2) in intermittent exotropia. Br J Ophthalmol.

[6] Hong SW, Park SC (2008). Development of distant stereoacuity in visually normal children as measured by the Frisby-Davisdistance stereotest. Br J Ophthalmol.

[7] Holmes JM, Fawcett SL (2005). Testing distance stereoacuity with the Frisby-Davis 2 (FD2) test. Am J Ophthalmol.

[8] Singh A, Sharma P, Singh D, Saxena R, Sharma A, Menon V (2013). Evaluation of FD2 (Frisby Davis distance) stereotest in surgical management of intermittent exotropia. Br J Ophthalmol.

[9] Garnham L, Sloper JJ (2006). Effect of age on adult stereoacuity as measured by different types of stereotest. Br J Ophthalmol.

[10] Lee JY, Seo JY, Baek SU (2013). The effects of glasses for anisometropia on stereopsis. Am J Ophthalmol.

[11] Tejedor J, Ogallar C (2008). Comparative efficacy of penalization methods in moderate to mild amblyopia. Am J Ophthalmol.

[12] Yang JW, Son MH, Yun IH (2004). A study on the clinical usefulness of digitalized random-dot stereoacuity test. Korean J Ophthalmol.

[13] Maeda M, Sato M, Ohmura T, Miyazaki Y, Wang AH, Awaya S (1999). Binocular depth-from-motion in infantile and late-onset esotropia patients with poor stereopsis. Invest Ophthalmol Vis Sci.

[14] Arnoldi K, Frenkel A (2014). Modification of the titmus fly test to improve accuracy. Am Orthopt J..

[15] Matsuo T, Negayama R, Sakata H, Hasebe K (2014). Correlation between depth perception by three-rods test and stereoacuity by distance RandotStereotest. Strabismus.

[16] Momeni-Moghaddam H, Eperjesi F, Kundart J, Mostafavi-Nam K (2014). Stereoacuity as an indicator of prism adaptation. Curr Eye Res.

[17] van Doorn LL, Evans BJ, Edgar DF, Fortuin MF (2014). Manufacturer changes lead to clinically important differences between two editions of the TNOstereotest. Ophthalmic Physiol Opt.

[18] Anketell PM, Saunders KJ, Little JA (2013). Stereoacuity norms for school-age children using the Frisby stereotest. J AAPOS.

[19] Kaye SB, Chen S (2003). Vertical and horizontal disparity with different orientations of the TNO stereotest. Optom Vis Sci.

[20] Han SB, Yang HK, Kim J, Hong K, Lee B, Hwang JM (2015). New stereoacuity test using a 3-dimensional display system in children. PLoS One..

[21] Jung JH, Yeom J, Hong J, Hong K, Min SW, Lee B (2011). Effect of fundamental depth resolution and cardboard effect to perceived depth resolution on multi-view display. Opt Express.

[22] Han J, Han SY, Lee SK, Lee JB, Han SH (2014). Real stereopsis test using a three-dimensional display with Tridef software. Yonsei Med J.

[23] Ma DJ, Yang HK, Hwang JM (2013). Reliability and validity of an automated computerized visual acuity and stereoacuity test in children using an interactive video game. Am J Ophthalmol.

[24] Westheimer G (2013). Clinical evaluation of stereopsis. Vision Res..

[25] Kim J, Yang HK, Kim Y, Lee B, Hwang JM (2011). Distance stereotest using a 3- dimensional monitor for adult subjects. Am J Ophthalmol.

[26] American Academy of Ophthalmology Pediatric Ophthalmology/Strabismus Panel. Preferred Practice Pattern Guidelines. Amblyopia. San Francisco, CA: American Academy of Ophthalmology; 2012. Available at: http://www.aao.org/preferred-practice-pattern/amblyopia-ppp--september-2012. Accessed 28 Sep 2012.

[27] Rutstein RP, Cogen MS, Cotter SA, Daum KM, Mozlin RL, Ryan JM. Care of the patient with Strabismus: Esotropia and Exotropia, American Optometric Association Optometric Clinical Practice Guideline; 2004. Available at: http://www.aoa.org/documents/optometrists/CPG-12.pdf. Accessed 27 Dec 2006.

[28] Kulp MT, Raasch TW, Polasky M. Patients with Anisometropia and Aniseikonia, In Benjamin, WJ (ed). Borish's Clinical Refraction. 2nd ed. St. Louis: Butterworth–Heinemann Elsevier; 2006:1479-1508.

[29] Chen JY, Oden RV, Merritt JO (2014). Utility of stereoscopic displays for indirect-vision driving and robot teleoperation. Ergonomics.

[30] Khairuddin HR, Malik AS, Mumtaz W, Kamel N, Xia L (2013). Analysis of EEG signals regularity in adults during video game play in 2D and 3D. Conf Proc IEEE Eng Med Biol Soc..

[31] Berkelman P, Miyasaka M, Bozlee S (2013). Co-located haptic and 3D graphic interface for medical simulations. Stud Health Technol Inform..

[32] Oliveira S, Jorge J, González-Méijome JM (2012). Dynamic accommodative response to different visual stimuli (2D vs 3D) while watching television and while playing Nintendo 3DS console. Ophthalmic Physiol Opt.

[33] Chen Y, Sun Y, Yang L, Wu F, Jiang XC, Liu S, Jin H, Wu H (2015). A new method to measure stereoacuity with 3D shutter glasses technology. Chin J Lab Diagn.

[34] Woo GC, Sillanpaa V (1979). Absolute stereoscopic thresholds as measured by crossed and uncrossed disparities. Am J Optom Physiol Opt.

[35] Grabowska A (1983). Lateral differences in the detection of stereoscopic depth. Neuropsychologia.

[36] Larson WL (1990). An investigation of the difference in stereoacuity between crossed and uncrossed disparities using Frisby and TNO tests. Optom Vis Sci.

[37] Jaschinski W, Schroth V (2008). Ocular prevalence: difference between crossed and uncrossed disparities of stereo objects. Strabismus.

[38] Saladin JJ. Phorometry and Stereopsis, In Benjamin,WJ. Borish's Clinical Refraction. Butterworth-Heinemann, 2nd edition, 2006: 899–960.

